# Leaf δ^15^N as a physiological indicator of the responsiveness of N_2_-fixing alfalfa plants to elevated [CO_2_], temperature and low water availability

**DOI:** 10.3389/fpls.2015.00574

**Published:** 2015-08-11

**Authors:** Idoia Ariz, Cristina Cruz, Tomé Neves, Juan J. Irigoyen, Carmen Garcia-Olaverri, Salvador Nogués, Pedro M. Aparicio-Tejo, Iker Aranjuelo

**Affiliations:** ^1^Faculdade de Ciências, Centro Ecologia Evolução e Alterações Ambientais, Universidade de LisboaLisboa, Portugal; ^2^Grupo de Fisiología del Estrés en Plantas, Departamento de Biología Ambiental, Unidad Asociada al CSIC, EEAD, Zaragoza e ICVVLogroño, Spain; ^3^Departamento de Estadística e Investigación Operativa, Universidad Pública de NavarraPamplona, Spain; ^4^Departamento de Biología Vegetal, Facultat de Biologia, Universidad de BarcelonaBarcelona, Spain; ^5^Departamento de Ciencias del Medio Natural, Universidad Pública de NavarraPamplona, Spain; ^6^Plant Biology and Ecology Department, Science and Technology Faculty, University of the Basque CountryLeioa, Spain; ^7^Instituto de Agrobiotecnología (IdAB), Universidad Pública de Navarra-CSIC-Gobierno de NavarraMutilva Baja, Spain

**Keywords:** alfalfa, climate change, growth, **δ**^15^N, physiology

## Abstract

The natural ^15^N/^14^N isotope composition (δ^15^N) of a tissue is a consequence of its N source and N physiological mechanisms in response to the environment. It could potentially be used as a tracer of N metabolism in plants under changing environmental conditions, where primary N metabolism may be complex, and losses and gains of N fluctuate over time. In order to test the utility of δ^15^N as an indicator of plant N status in N_2_-fixing plants grown under various environmental conditions, alfalfa (*Medicago sativa* L.) plants were subjected to distinct conditions of [CO_2_] (400 vs. 700 μmol mol^−1^), temperature (ambient vs. ambient +4°C) and water availability (fully watered vs. water deficiency—WD). As expected, increased [CO_2_] and temperature stimulated photosynthetic rates and plant growth, whereas these parameters were negatively affected by WD. The determination of δ^15^N in leaves, stems, roots, and nodules showed that leaves were the most representative organs of the plant response to increased [CO_2_] and WD. Depletion of heavier N isotopes in plants grown under higher [CO_2_] and WD conditions reflected decreased transpiration rates, but could also be related to a higher N demand in leaves, as suggested by the decreased leaf N and total soluble protein (TSP) contents detected at 700 μmol mol^−1^ [CO_2_] and WD conditions. In summary, leaf δ^15^N provides relevant information integrating parameters which condition plant responsiveness (e.g., photosynthesis, TSP, N demand, and water transpiration) to environmental conditions.

## Introduction

Considering the current rate of increase in CO_2_ emissions (1.5 μmol mol^−1^ year^−1^), it is expected that atmospheric CO_2_ concentrations ([CO_2_]) will reach 550 μmol mol^−1^ by 2050 and 700 μmol mol^−1^ by 2100 (Myhre et al., 2013). The associated warming is expected to be greatest in summer in south-western Europe.

Although atmospheric [CO_2_] is now limiting for C_3_ photosynthesis and growth, the predicted increase in [CO_2_] in coming decades could enhance photosynthetic rates and biomass production in C_3_ plants (Farquhar et al., 1980a; Bowes, 1993; Amthor, 2001; Long et al., 2004). Nevertheless, the interaction of CO_2_ with other limiting environmental factors, (e.g., higher temperature, lower water, and/or nitrogen availabilities) might decrease or eliminate the positive effect of elevated CO_2_ on plant production (Ainsworth et al., 2004; Rogers et al., 2009; Aranjuelo et al., 2011).

Most experiments analysing the effects of climate change on plant growth have considered the variability of individual environmental factors (CO_2_, temperature, water availability), keeping others at levels optimal for growth. However, analyses of the effect of CO_2_ and its interaction with other environmental conditions are of great relevance. In the field, multiple stresses, such as high temperature and drought periods in semi-arid or drought-stricken areas, often occur simultaneously. Studies of field crops and model plants have shown that the combination of heat and drought stresses has a stronger detrimental effect on plants growth and productivity than either stress alone. Furthermore, many reports indicate that it is not possible to extrapolate plant responses to combined stresses based on the responses to single stresses (Rampino et al., 2012).

In recent decades, stable isotope techniques (Isotope Ratio Mass Spectrometry, IRMS, mostly with ^13^C and ^18^O) have been used as tools that provide useful information on parameters conditioning plant growth, such as transpiration efficiency, the ratio of net photosynthesis to water transpired, etc., and that integrate the period during which CO_2_ is assimilated (Araus et al., 2002, 2003; Yousfi et al., 2010). Moreover, ^13^C isotope composition (δ^13^C) has been used as a breeding criterion for increasing yield in crops exposed to low water availability and salinity stresses (Yousfi et al., 2009, 2010; Araus et al., 2013). Variations in ^15^N isotopic composition (δ^15^N) have also been proposed as a useful trait for crop screening (Pritchard and Guy, 2005; Yousfi et al., 2012). Robinson et al. (2000) proposed that the natural abundance of both ^13^C and ^15^N might indicate responses to stresses such as drought and nitrogen starvation. Moreover, δ^13^C and δ^15^N have been used to characterize the response of crops to salinity (Yousfi et al., 2009) and are widely used in plant ecophysiology to assess the effects of changing climatic conditions as both are sensitive to environmental constraints (Peuke et al., 2006). Three main factors have been described (Evans, 2001; Pritchard and Guy, 2005; Coque et al., 2006; Tcherkez, 2011) as determining plant δ^15^N: (i) morphophysiological differences (particularly in root systems); (ii) activity of principal enzymes involved in N assimilation and (iii) plant N demand and assimilation capacity. However, it should be remembered that further ^15^N fractionation might take place as a result of N recycling, transport, exudation or volatilization (through stomata as ammonia and nitrous oxide) by the plants (Cernusak et al., 2009). Although δ^15^N has been previously determined in N_2_-fixing plants (Arnone, 1999; Wanek and Arndt, 2002), with very few exceptions (Shearer et al., 1982; Unkovich, 2013) this parameter has been mostly determined in plants grown with both N sources: N_2_ and NO^−^_3_. The natural ^15^N abundance method has been widely used to provide semi-quantitative estimates of the relative contribution of atmospheric N_2_ to N_2_-fixing plants growing in natural and agricultural settings (Shearer and Kohl, 1988), where N is available in several forms (i.e., NO^−^_3_, NH^+^_4_, N_2_, etc.). Thus, despite recent advances in the interpretation of plant δ^15^N, there is still a lack of knowledge of δ^15^N in plants where N_2_-fixation is the sole source of N.

Given that atmospheric N_2_ is an unlimited N source, and that N_2_-fixing legumes comprise the second most important group of agricultural crops worldwide (FAOSTAT, 2010^1^), the use of δ^15^N as an integrative indicator of the responsiveness of N_2_-fixing plants to climate change conditions may be of great interest. The study of δ^15^N gradients along plant axes (from N source to sinks) and their reaction to environmental stresses may provide valuable information on the transport and metabolism of C-N compounds (Peuke et al., 2006). To achieve this, exclusively N_2_-fixing alfalfa (*Medicago sativa* L.) plants, which are frequently exposed to high temperature and/or drought in field conditions, were studied. They were subjected to distinct levels of [CO_2_] (400 vs. 700 μmol mol^−1^), temperature (ambient vs. ambient +4°C) and water availability (fully watered vs. partially watered). In addition to growth, we characterized the N isotopic composition (δ^15^N) of whole plants and separate organs (leaves, stems, roots and nodules), and δ^15^N relationship with C-N related parameters.

## Materials and methods

### Plant material and experimental design

Alfalfa (*Medicago sativa* L. cv Aragon) plants were grown in 13 L plastic pots (five plants per pot) filled with 1:2 (v/v) vermiculite-perlite. At 2–4 weeks after planting, they were inoculated with *Sinorhizobium meliloti* strain 102F78 (The Nitragin Co., Milwaukee, WI, USA). One-month-old plants were transferred to the corresponding temperature gradient greenhouses (TGG; Figure S1). The experimental design and the use of the greenhouses were similar to that described by Morales et al. (2014). Half of the plants were placed at 700 μmol mol^−1^ of [CO_2_] in a TGG, whereas the other half was grown in a different TGG under ambient [CO_2_] (400 μmol mol^−1^). Within each TGG, one for each CO_2_ concentration (400- and 700-μmol mol^−1^), plants were separated into 4 treatments corresponding to all combinations of, temperature (ambient—around 19°C—and ambient +4°C) and water availability (control –fully irrigated- or drought -partially irrigated-). After 1 month development, at the corresponding growth conditions, gas exchange measurements and harvest were carried out (60 days—old plants).

#### [CO_2_] control within the TGGs

Ventilated [CO_2_] temperature and humidity sensors (M22W2HT4X transmitters, Rotronic Instrument Corp., Hauppauge, USA) and air probes connected to another CO_2_ infrared gas analyser were placed at the center of each module 60 cm above the plants.

The [CO_2_], concentration was monitored continuously at the outlet module by an infrared analyser (Guardian Plus gas monitor, Edinburgh Instruments Ltd, Livingston, UK) whose signal was fed into a proportional integrative differential controller that regulated the opening time (within a 10-s cycle) of a solenoid valve that injected CO_2_ into both inlet fans because otherwise lateral mixing of CO_2_ in the chambers was not complete. The data were continuously recorded by a computer through analog-digital converters (Microlink 751, Biodata Ltd, Manchester, UK) using Windmill software with the Test-Seq programming tool (Biodata Ltd). A subroutine of this software controlled solenoid valves that kept one of two sets of CO_2_ cylinders open or closed (provided by Air Liquide, Bilbao, Spain) thus supplying the gas to the elevated CO_2_ tunnel. When CO_2_ concentration decreased below a fixed level, signaling that one of the cylinder sets was exhausted, the corresponding valve was closed and that of the other set opened.

#### Temperature control within the TGGs

The measured temperature difference was used to set the required fan speed by altering the current: the gradient decreased or increased as the fan was sped or slowed, respectively. Two inlet fans (each 90 W, 0.5 m^3^ s^−1^) mounted on the inlet module and an outlet fan (140 W, 0.54 m^3^ s^−1^) mounted in the roof of the outlet compartment continuously circulated air through the tunnel at the speed required to maintain a difference of 4°C between the two extreme modules. The fan at the tunnel outlet was in the roof, rather than in the end wall of the outlet compartment, so that any external wind would not disrupt the temperature gradient (Morales et al., 2014). Air flow was continuously varied by changing the fan speed to achieve the end-to-end temperature difference. Three small fan heaters (variable 250–500 W), placed above plant level in the outlet compartment and facing the tunnel interior, were used to help maintain the temperature difference at night and whenever solar radiation was insufficient to raise the temperature.

#### Water treatment

When analysing the interaction between [CO_2_] and water availability, it should be remembered that plants grown at elevated [CO_2_] deplete soil water at a lower rate than those grown with ambient [CO_2_] (due to lower stomatal conductance and lower transpiration rates), so in many experiments, elevated [CO_2_] increased the time to reach a particular water stress (De Luis et al., 1999; Aranjuelo et al., 2009). To test this, we designed an experiment in which all treatments were subjected to the same soil water content. Well-watered (WW) plants were irrigated until they reached maximum soil volumetric water content (θ_v_), whereas partially irrigated plants (WD) were watered at 50% θ_v_ of WW plants. These θ_v_ levels were maintained throughout the experiment by daily measurement of transpired water (calculated by weighing the pots) and replenishing the lost water. In order to reduce evaporation from the soil, pots were covered with a plastic sheet perforated with very small holes to allow stems to pass through. In order to supply all treatments with the same amount of nutrients, WW plants were alternately watered with Evans N-free nutrient solution and distilled water, while WD plants were always watered with Evans solution. Pots were rotated weekly in each module to avoid edge effects. In order to avoid differences due to chamber effects, the plants were moved from one greenhouse to another every month. All the determinations listed below were made at the end of the experiment, when the plants were 60 days old, in apical fully expanded leaves.

### Plant growth determinations

Plant growth in the TGGs under the aforementioned [CO_2_], temperature and water availability conditions was determined by harvesting after 1 month of growth. Twenty plants were collected per treatment combination. The plants were divided into leaves, stems, roots and nodules, and the fresh weight of these components was recorded. After drying at 60°C for 48 h, their dry weight was determined. Leaf area was analyzed with an electronic planimeter (Li-3000 with LI-3050 conveyer accessory, LICOR, NE, USA). Total dry matter (DM) comprised leaf, stem, root and nodule DM.

### Total soluble protein (TSP) content

Proteins were extracted from frozen leaf subsamples and ground to a fine powder [in 50 mM Tricine buffer, pH 8.0, 1 mM EDTA, 5 mM 6-aminocaproic acid, 2 mM benzamidine, 8 mM β-mercaptoethanol, and 100 mM phenylmethylsulfonylfluoride (PMSF)]. This was kept on ice for 20 min and then centrifuged at 12,000 g and 4°C for 25 min. The total soluble protein content of the supernatant was determined according to the Bradford method (Bradford, 1976).

### Gas exchange analyses

Fully expanded apical leaves from 50-day-old plants were individually enclosed in a leaf chamber (1010-M, Waltz, Effeltrich, Germany), and the gas exchange rate was measured with a portable photosynthesis system (HCM-1000, Waltz) under growth conditions. Net photosynthesis (A) and leaf conductance (g) were calculated as described by von Caemmerer and Farquhar (1981). The leaf internal CO_2_ concentration (Ci) was estimated from net photosynthesis and conductance measurements according to Farquhar and Sharkey (1982). Fully expanded leaves were enclosed in a GFS-3000 portable gas exchange system (Walz, Effeltreich, Germany). Gas exchange analyses were conducted in every plant grown at 400 and 700 μmol mol^−1^ [CO_2_] (A_400_ and A_700_ respectively), at the corresponding growth temperature and with a photosynthetic photon flux density of 1200 μmol m^−2^ s^−1^.

### C and N isotope and content analysis

A subsample of frozen leaf, stem, root and nodule from each plant was dried at 60°C for 48 h in small tin capsules and weighed. The nitrogen and carbon isotope composition of the samples was determined using a Flash 1112 Elemental Analyzer (Carbo Erba, Milan) coupled to an IRMS Delta C isotope ratio mass spectrometer through a Conflo III Interface (Thermo-Finnigan, Germany).

Nitrogen results were expressed in parts per thousand (‰) in the δ notation (δ^15^N) using international secondary standards of known ^15^N/^14^N ratios (IAEA N_1_ and IAEA N_2_ ammonium sulfate and IAEA NO_3_ potassium nitrate) relative to N_2_ in air:

(1) $$\delta^{15}\text{N}=\left(\frac{R_{\text{sample}}}{R_{\text{standard}}}\right)-1$$

where *R* is the ^15^N/^14^N ratio.

N and C contents were determined in three biological replicates of dried nodule, root and leaf samples, ground to powder, weighed (1.0 mg per sample) and stored in tin capsules. N and C content were determined at the Serveis Cientifico-Técnics of the University of Barcelona (Barcelona, Spain) using an elemental analyser (EA1108, Series 1; Carbo Erba Instrumentazione, Milan, Italy).

#### Statistical analysis

Statistical analyses were performed with the programs SPSS for Windows, version 15.0 (Sections Statistical analysis of physiological and C-N-related parameters in N_2_-fixing alfalfa plants grown under various environmental conditions and Regression analyses of axial patterns of δ^15^N) and Statistica 10, data analysis software system, version 10 (StatSoft, Inc. 2011; Section Statistical analyses of leaves: relationships among C-N natural isotopic abundances and physiological parameters.).

##### Statistical analysis of physiological and C-N-related parameters in N_2_-fixing alfalfa plants grown under various environmental conditions

We examined results from eight treatments using analysis of variance (ANOVA) to test for effects and interactions of the various combinations of three environmental factors ([CO_2_], temperature and water availability), and whether these results varied according to the organ tested. Besides analysis of whole plants (exploratory analysis, data not shown), each organ (nodule, root, stem, and leaves) was analyzed separately. Homoscedasticity was determined using the Levene test (Levene, 1960), then One- and Two-Way ANOVA tests, including interaction terms, were conducted using data displayed in **Figure 2**, **Tables 2, 3** and Tables S1–S3.

##### Regression analyses of axial patterns of δ^15^N

Linear regression models (Table 1) were performed using the model: Y = (a) + bX, where Y corresponds to δ^15^N_sink−organ_ and X corresponds to δ^15^N_source−organ_.

**Table 1:** **Regression analysis between organ δ^15^N in 60-day-old nodulated alfalfa plants exposed to differing climate conditions**.

**Factor**		**[CO_2_] (μmol CO_2_ mol^−1^)**		**Temperature (°C)**		**Water availability**		**Global**
		**400**	**700**	**Ambient**	**+ 4**	**WW**	**WD**	
Stem/Leaves	Slope	0.7^*^	0.99	1.33^*^	0.76	1.02^***^	1.28^**^	1.10^**^
	*p*-value	**0.072**	ns	**0.089**	ns	**0.009**	**0.024**	**0.021**
Root/Stem	Slope	−0.83^**^	0.63	0.80	−0.39	−0.40	1.19	−0.31
	*p*-value	**0.011**	ns	ns	ns	ns	ns	ns
Nodule/Root	Slope	−0.2	−0.06	−0.26	−0.14	−0.77^**^	−0.03	−0.39^*^
	*p*-value	ns	ns	ns	ns	**0.034**	ns	**0.084**

**Figure 1:**
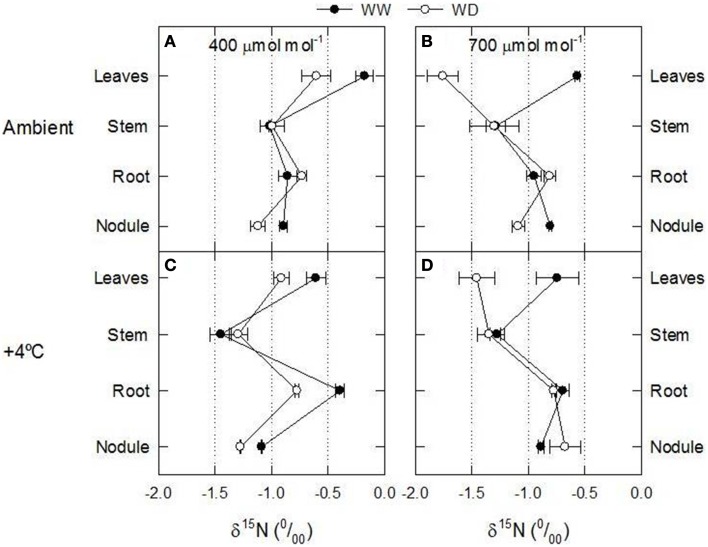
**Axial patterns of δ^15^N in 60-day-old nodulated alfalfa plants grown under differing conditions of CO_2_ concentration [400 μmol CO_2_ mol^−1^, left panels (A,C), or 700 μmol CO_2_ mol^−1^, right panels (B,D)], temperature [ambient, upper panels (A,B), or +4°C, lower panels (C,D)] and water availability (well watered, WW, or water deficiency, WD)**. This figure summarizes data concerning δ^15^N values showed in **Table 4** and Tables S1–S3. Data represent average values ± SE (*n* = 3).

Nodule against root; root against stem; stem against leaves. The slopes from linear regression models [Model: Y = (a) + bX, where Y corresponds to δ^15^N_sink−organ_ and X corresponds to δ^15^N_source−organ_] are given with p-values and significances (ns, no significant differences, p > 0.1;

* refer to significant differences where P ≤ 0.1;

** refer to significant differences where P ≤ 0.05;

*** refer to significant differences where P ≤ 0.01;

^****^refer to significant differences where P ≤ 0.001). Significant values are shown in bold text. For further details see legend to Figure 1.

##### Statistical analyses of leaves: relationships among C-N natural isotopic abundances and physiological parameters

Following an exploratory-inferential approach, data analysis revealed that leaves were the organs that were the most influenced by environmental factors, so several descriptive statistical analyses were conducted only on data from leaves. Simple regression models were estimated for δ^15^N and target parameters conditioning plant growth (e.g., plant biomass, plant level photosynthesis, TSP, leaf area, N content). Correlation and simple regression models for leaf parameters (**Figures 3–5**) were used to determine R^2^ and *p*-values for each analysis.

The results of this study were obtained for plants cultured in several independent series, at least one sample was analyzed for each of three independent series. Sample size varied depending on the analysis carried out, from 32 (for organ specific descriptive analysis) up to 192 (for exploratory-inferential analysis).

## Results and discussion

It is generally accepted that leaf δ^15^N reflects the ^15^N abundance of plant main N source(s): available soil N for non-N_2_-fixing plants and atmospheric N_2_ for N_2_-fixing plants (Shearer and Kohl, 1988). Since, by definition, the δ^15^N of atmospheric N_2_ is 0, that of N_2_-fixing plants growing without any other N source should also be around 0, but in fact it can be very distinct from zero (Unkovich, 2013). The precise value of the N_2_-fixing plants δ^15^N depends, among other factors, on: (1) the physiological partition of the N metabolism between shoot and root; (2) the N efflux; and (3) on the exudation of metabolites. The δ^15^N values of the distinct plant organs (nodules, roots, stems and leaves) show that leaf δ^15^N is the one more responsive to environmental factors (Figure 1; Table 1). The increase of ambient temperature by 4°C, did not significantly modify any leaf C-N related parameter (including leaf δ^15^N; Tables 1–**3**). The combined effect of increased [CO_2_] and WD caused the more significant changes in leaf δ^15^N (Figure 1, Table 1).

### δ^15^N as affected by stomatal opening

**Figure 2:**
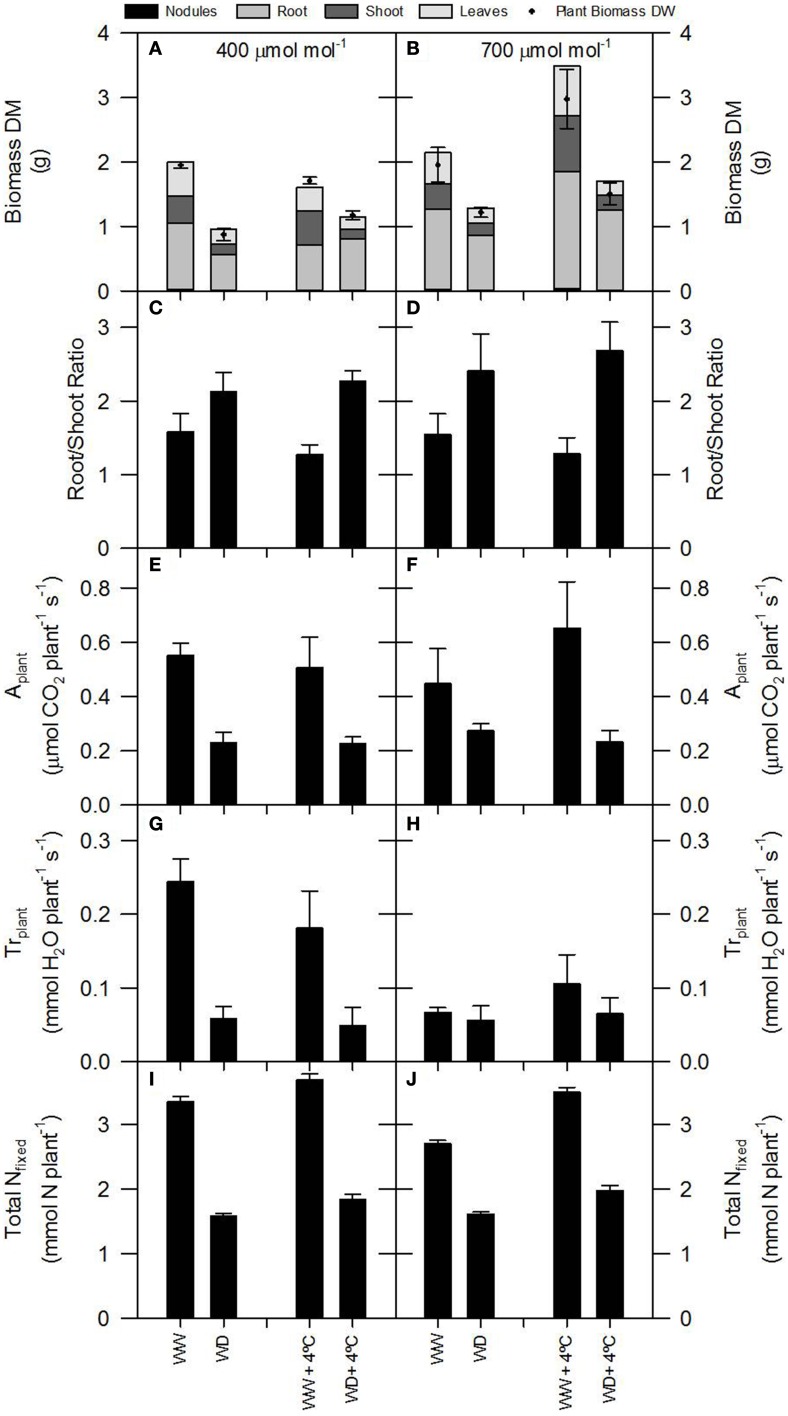
**Plant growth (dry matter, DM, and root/shoot ration), total photosynthesis, total transpiration and total N fixed per plant of 60-day-old nodulated alfalfa plants exposed to differing environmental conditions: CO_2_ concentration (400 μmol CO_2_ mol^−1^, left panels, or 700 μmol CO_2_ mol^−1^, right panels); temperature (ambient or +4°C); and water availability (well watered, WW, or water deficient, WD). Legend: (A,B)—relative and total plant growth; the relative bar areas represent the individual organ percentage relative to the total plant growth (black line); (C,D)—root/shoot ratio; (E,F)—total photosynthesis, *A*_*plant*_; (G,H)—total transpiration, *Tr*_*plant*_; (I,J)—total N fixed**. Data represent average values ± SE (*n* = 3–6).

**Figure 3:**
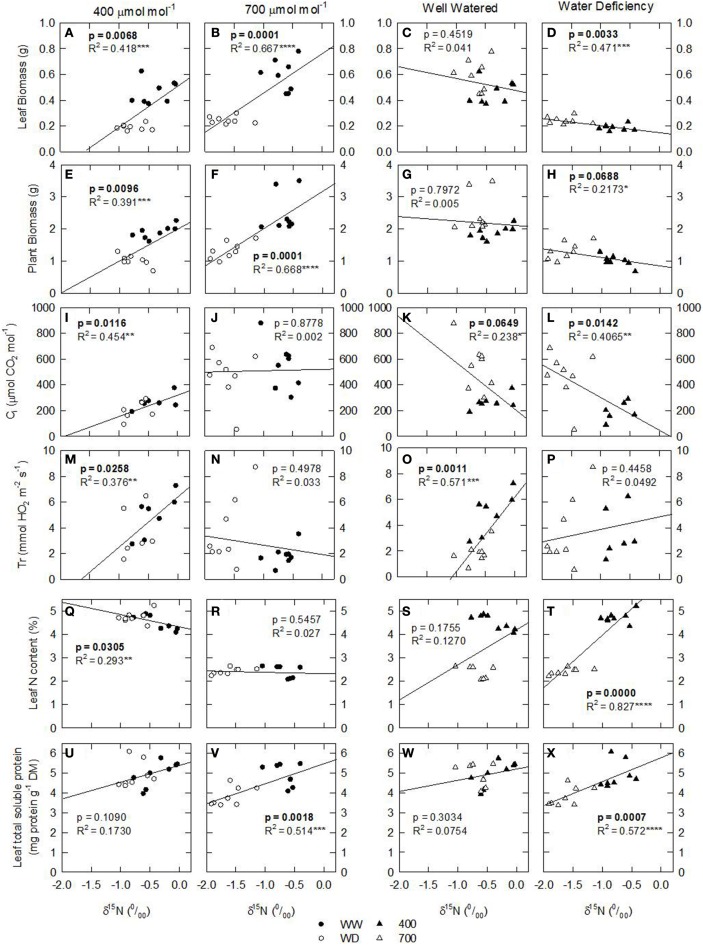
**Leaf N isotopic composition (δ^15^N; ‰) of nodulated alfalfa plants exposed to differing environmental conditions correlated with: (A–H) leaf biomass (grams); (I–L) C_i_ (μmol CO_2_ mol^−1^); (M–P) transpiration rate (mmol H_2_O m^−2^ s^−1^); (Q–T) leaf N content (%, w/w); and (U–X) leaf total soluble protein (TSP; mg prot g^−1^ DM)**. Datasets were categorized in terms of environmental conditions: [CO_2_], 400 or 700 ppm, left panels; water availability, well watered—WW, or water deficient—WD, right panels. The dataset displayed represents individual observations, at least *n* = 3 for each environmental combination. Significant *p*-values are shown in bold text. Significance: *p* > 0.1; ^*^P ≤ 0.1; ^**^P ≤ 0.05; ^***^P ≤ 0.01; ^****^P ≤ 0.001.

Control plants (400 ppm CO_2_, WW, environmental temperature) did not show differences between the δ^15^N values of nodules, roots or stems (δ^15^N ≈ −1.0), while leaves presented δ^15^N values closer to zero (Figure 1A). Theoretically this relative enrichment of the leaves in ^15^N may be due to NH_3_ losses through stomata (Farquhar et al., 1980b), and may be associated with two main factors: (1) the leaf NH_3_ pool is predominantly originated through photorespiration and may have a δ^15^N as low as −40 (Handley et al., 1999; Peuke et al., 2006); and (2) the ^14^N is lost more readily through the stomata than ^15^N (O'Deen, 1989). In fact both environmental factors, [CO_2_] and water availability lead to reduced stomatal conductance (Figures S2E,F) and transpiration rates (Figures 2G,H; Figures S2C,D; Table 2). As a consequence, the δ^15^N of leaves from plants grown at increased [CO_2_] and/or WD tended to have lower leaf δ^15^N than those from plants grown at ambient [CO_2_] or from WW plants (i.e., higher stomatal opening Figures 1, 3). However, the ranges of ^15^N depletion in leaves caused by both factors, WD and [CO_2_], were not exactly the same (≈−0.5 for [CO_2_] and ≈−1 to −1.5 for WD; Figure 1).

**Table 2:** **Analysis of variance of the effect of [CO_2_], water availability and temperature on plant growth, gas exchange and N fixation parameters**.

**Factor**	**Total biomass DM (g)**	**Root/Shoot Ratio**	**A_plant_ (μmol CO_2_ plant^−1^ s^−1^)**	**Tr_plant_ (mmol H_2_O plant^−1^ s^−1^)**	***g*_growth_ (mmol CO_2_ m^−2^ s^−1^)**	**Total N_fixed_ μmol N_fixed_ plant^−1^**
[CO_2_]	^*^	ns	ns	^*^	^***^	ns
H_2_O	^****^	^****^	^****^	^***^	ns	^****^
T	ns	ns	ns	ns	ns	ns
[CO_2_]^*^H_2_O	−	−	−	+	ns	+
[CO_2_]^*^T	−	−	−	−	ns	−
H_2_O^*^T	−	−	−	−	ns	−

The effects of carbon dioxide concentration ([CO_2_]), water availability (H_2_O), temperature (T) and their peer interactions ([CO_2_] ^*^ H_2_O; [CO_2_] ^*^ T and H_2_O ^*^ T) were determined by (One- and Two-Way) ANOVA tests using SPSS software. Significant effects are shown with asterisks (

* refer to significant differences where P ≤ 0.1;

^**^refer to significant differences where P ≤ 0.05;

*** refer to significant differences where P ≤ 0.01;

**** *refer to significant differences where P ≤ 0.001; interaction between factors, +; no interaction between factors, −). Letters ns denote no significant differences (n = 3)*.

### Leaf δ^15^N as an indicator of plant N demand and organ N partitioning

**Figure 4:**
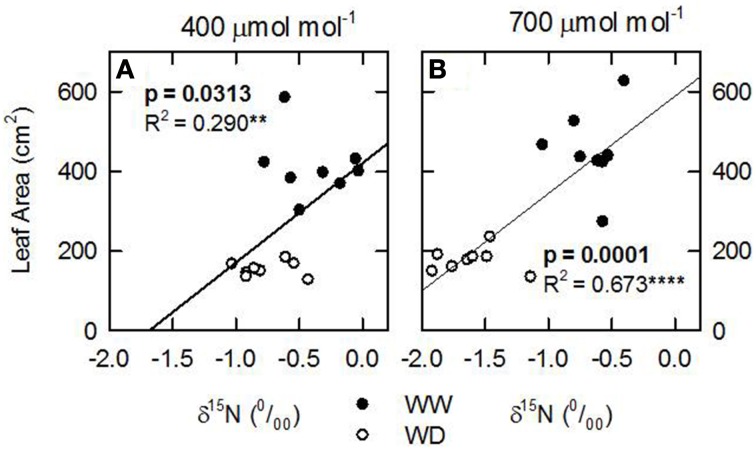
**Leaf N isotopic composition (δ^15^N; ‰) of nodulated alfalfa plants exposed to differing environmental conditions correlated with (A,B) leaf area (cm^2^)**. The dataset was categorized by [CO_2_], 400, left panels or 700 ppm, right panels. Legend for water availability treatments: well watered, WW; or water deficient, WD. The dataset displayed represents individual observations, at least *n* = 3 for each environmental combination. Significant *p*-values are shown in bold text. Significance: *p* > 0.1; ^*^refer to significant differences where *P* ≤ 0.1; ^**^refer to significant differences where *P* ≤ 0.05; ^***^refer to significant differences where *P* ≤ 0.01; ^****^refer to significant differences where *P* ≤ 0.001.

**Figure 5:**
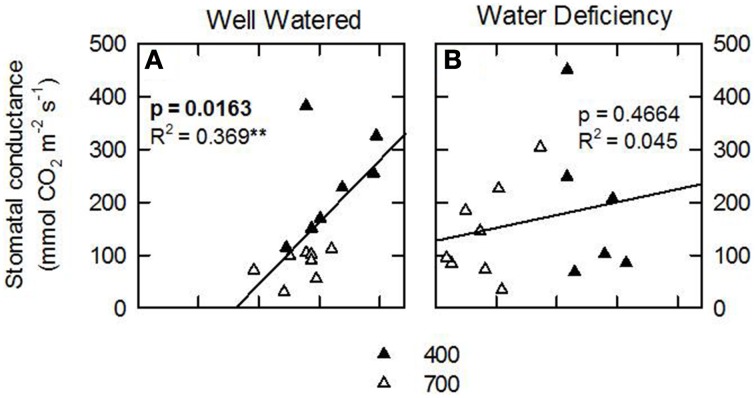
**Leaf N isotopic composition (δ^15^N; ‰) of nodulated alfalfa plants exposed to differing environmental conditions correlated with stomatal conductance (mmol CO_2_ m^−2^ s^−1^)**. The dataset was categorized by water availability: well watered, WW, left panels; or water deficient, WD, right panels. Legend for [CO_2_] treatments, 400 or 700 ppm. The dataset displayed represents individual observations, at least *n* = 3 for each environmental combination. Significant *p*-values are shown in bold text. Significance: *p* > 0.1; ^*^refer to significant differences where *P* ≤ 0.1; ^**^refer to significant differences where *P* ≤ 0.05; ^***^refer to significant differences where *P* ≤ 0.01; ^****^refer to significant differences where *P* ≤ 0.001.

Considering that the variability of δ^15^N in leaves reflects changes in N metabolic and metabolite fluxes, and/or environment-driven effects, leaf δ^15^N has been proposed as a good candidate for tracing these effects in plants (Tcherkez, 2011). Plants showing healthy physiological features (i.e., higher leaf and plant biomass, leaf area, leaf N content, and leaf TSP) had leaf δ^15^N values closer to that of their N source (δ^15^N_atmosphere_ = 0; Figures 1–3). In contrast, plants affected by [CO_2_] and water availability, with impaired growth (Figure 2, Table 2), had more negative leaf δ^15^N values (−2 to −0.5; Figure 1). These differences highlight the effect of environmental factors on transport and partitioning of N metabolism in N_2_-fixing plants (Peuke et al., 2006). Correlation-regression analyses confirmed that both environmental factors ([CO_2_] and water availability) influenced the correlations between leaf δ^15^N and biomass and several physiological parameters (leaf biomass, plant biomass, internal concentration of CO_2_, transpiration, foliar N content and foliar TSP) (Figure 3). However, some other relationships were mostly influenced by [CO_2_] (e.g., leaf area; Figure 4) or by water availability (e.g., stomatal conductance; Figure 5). The depletion of foliar δ^15^N under high [CO_2_] has also been observed in a wide range of plant species (27 field-grown plant species) and ecosystem types (Bassirirad et al., 2003). However, there is no direct evidence that water availability influences foliar N isotope composition (Peuke et al., 2006).

This differential response of leaf δ^15^N to the combination of [CO_2_] and water availability, together with the observed low correlations between leaf δ^15^N and plant transpiration associated with high [CO_2_] and water deficiency (Figures 3N,P), suggest that other metabolic processes (different from stomatal conductance, see above) could be involved in such an isotopic effect. Higher [CO_2_] and WD led to different C/N balances and N requirements (Figures 2I,J; Tables 3, 4), which may be related to the observed differences in leaf δ^15^N. Despite the potentially increased C availability at higher [CO_2_], and the higher plant growth demonstrated by these plants (Figure 2; Table 2), they did not increase their total fixed-N_2_ (Figure 2), leading to unbalanced foliar N contents (%; ≈ 2% at 700 vs. ≈ 4–5% at 400 μmol mol^−1^) and C/N ratios (Table 4). The lower foliar N content at higher [CO_2_] indicates a higher N demand, limiting plant growth under such conditions. This concept is supported by the similarity of the δ^15^N in leaves and nodules (WW plants, Figure 1), which suggests negligible losses of N and optimization of the N use efficiency (NUE) of the N_2_-fixing plants grown at high [CO_2_]. In other words, all fixed N is being used by the plants. In fact, plants containing increased leaf TSP contents had leaf δ^15^N values close to zero (δ^15^N_atmosphere_ = 0; Figure 3), so the growth of N_2_-fixing plants exposed to higher [CO_2_] is determined by their N_2_ fixation capacity. Similar results were described by Bassirirad et al. (2003) with mycorrhizal plants exposed to elevated [CO_2_].

**Table 3:** **Analysis of variance of the effect of [CO_2_], water availability and temperature on leaf C-N-related parameters**.

**Factor**	**Leaf area (cm^2^)**	**TSP (mg prot g^−1^ DM)**	**N content (%)**	**C/N**	**δ^15^N (‰)**
[CO_2_]	ns	^****^	^****^	^****^	^****^
H_2_O	^****^	^**^	ns	ns	^****^
T	ns	ns	ns	ns	ns
[CO_2_]^*^H_2_O	−	+	−	−	+
[CO_2_]^*^T	−	+	−	+	−
H_2_O^*^T	−	−	−	−	−

The effects of carbon dioxide concentration ([CO_2_]), water availability (H_2_O), temperature (T) and their peer interactions ([CO_2_]^*^H_2_O; [CO_2_]^*^T and H_2_O^*^T) were determined by (One- and Two-Way) ANOVA tests using SPSS software. Significant effects are shown with asterisks (

* refer to significant differences where P ≤ 0.1;

** refer to significant differences where P ≤ 0.05;

^***^refer to significant differences where P ≤ 0.01;

**** *refer to significant differences where P ≤ 0.001; interaction between factors, +; no interaction between factors, −). Letters ns denote, no significant differences (n = 3)*.

**Table 4:** **Responsiveness of leaf C-N-related parameters of 60-day-old nodulated alfalfa plants exposed to different climate conditions**.

**Treatments (CO_2_-H_2_O-T)**	**Leaf area (cm^2^)**	**TSP (mg prot g^−1^ DM)**	**N content (%)**	**C/N**	**δ^15^N (‰)**
400–WW–Amb	399 ± 13	5.3 ± 0.12	4.2 ± 0.06	10.8 ± 0.11	−0.14±0.06
400–WD–Amb	158 ± 12	5.5 ± 0.23	4.8 ± 0.18	9.8 ± 0.35	−0.60±0.09
400–WW–+ 4°C	423 ± 60	4.6 ± 0.19	4.8 ± 0.03	9.8 ± 0.07	−0.61±0.06
400–WD–+ 4°C	149 ± 7	4.6 ± 0.11	4.6 ± 0.02	10.2 ± 0.05	−0.91±0.05
700–WW–Amb	390 ± 39	4.5 ± 0.11	2.1 ± 0.01	20.8 ± 0.10	−0.57±0.02
700–WD–Amb	171 ± 10	3.6 ± 0.09	2.3 ± 0.05	19.5 ± 0.40	−1.76±0.10
700–WW–+ 4°C	513 ± 42	5.4 ± 0.04	2.6 ± 0.01	17.4 ± 0.01	−0.74±0.13
700–WD–+ 4°C	183 ± 21	4.2 ± 0.18	2.5 ± 0.07	18.5 ± 0.49	−1.45±0.11

*Parameters: leaf area (cm^2^); leaf total soluble proteins (TSP, mg prot g^−1^ DM); leaf N content (%, m/m); leaf C/N ratio; and N natural isotopic signature of leaves (‰). Environmental conditions: CO_2_ concentration (400 or 700 μmol CO_2_ mol^−1^), temperature (ambient, Amb, or 4°C) and water availability (well watered, WW, or water deficient, WD). Data represent average values ± SE (n = 3–6)*.

Plant N demand has been described as a key factor conditioning δ^15^N (Tcherkez, 2011), so the higher N demand by alfalfa leaves exposed to higher [CO_2_] could lead to differential N partitioning between the plant's above- and below-ground parts. On the other hand, translocation of organic N compounds rather than inorganic N (i.e., ammonium) from bacteroids to the plant (nodules, roots, stems, and finally leaves, mainly in the form of Asn in alfalfa plants; Kaspar et al., 2008) could also lead to a more ^14^N-enriched signature of plant organs, because the assimilated N organic pool in plants is generally^14^N-enriched relative to the unassimilated N inorganic pool (Werner and Schmidt, 2002; Kalcsits and Guy, 2013).

## Conclusion

Leaf δ^15^N was a sensitive integrator of such combined environmental stresses on N_2_-fixing alfalfa plants: plants affected by higher [CO_2_] and water deficiency, which displayed impaired growth features, had more negative leaf δ^15^N values than that of atmospheric N_2_. In contrast, physiologically healthy plants had leaf ^15^N signatures close to those of their N source (δ^15^N_atmosphere_ = 0). This observation, together with further investigation of isotope fractionation during transport and metabolic processes, may provide useful information on the metabolism, transport and allocation of N in N_2_-fixing plants exposed to combined environmental stresses.

### Conflict of interest statement

The authors declare that the research was conducted in the absence of any commercial or financial relationships that could be construed as a potential conflict of interest.

## Footnotes

^1^http://faostat.fao.org/site/291/default.aspx
