# Supplementary material for: Leaf δ15N as a physiological indicator of the responsiveness of N2-fixing alfalfa plants to elevated [CO2], temperature and low water availability
Source: Front Plant Sci. 2015 Aug 11;6:574. doi: 10.3389/fpls.2015.00574 (PMC4531240; doi:10.3389/fpls.2015.00574)
Supplement: Supplementary file 1 [file Table1.DOC]

**Table S1.** **Responsiveness of stem C-N-related parameters from 60-days-old nodulated-alfalfa plants exposed to different climate change conditions.** Parameters: N content (%, m/m), C/N ratio and N natural isotopic signature (‰) of stems. Environmental conditions: CO_2_ concentration (400 or 700 µmol CO_2_ mol^-1^), temperature (ambient, Amb, or +4ºC) and water availability (well watered, WW, or water deficiency, WD). Data represent average values±SE (n = 3).

| Treatments | N Content | C/N | δ^15^N |
| --- | --- | --- | --- |
| (CO_2_-H_2_O-T) | (%) |  | (‰) |
| 400 - WW - Amb | 1.6 ± 0.03 | 27.4 ± 0.50 | -1.01 ± 0.01 |
| 400 – WD - Amb | 1.9 ± 0.06 | 23.2 ± 0.75 | -0.99 ± 0.11 |
| 400 - WW - +4ºC | 1.6 ± 0.03 | 27.4 ± 0.10 | -1.45 ± 0.10 |
| 400 - WD - +4ºC | 1.9 ± 0.03 | 23.1 ± 0.38 | -1.30 ± 0.08 |
| 700 - WW - Amb | 1.6 ± 0.06 | 27.1 ± 0.40 | -1.29 ± 0.08 |
| 700 – WD - Amb | 1.4 ± 0.04 | 32.6 ± 0.94 | -1.30 ± 0.22 |
| 700 - WW - +4ºC | 1.4 ± 0.04 | 32.7 ± 0.66 | -1.28 ± 0.06 |
| 700 - WD - +4ºC | 1.4 ± 0.01 | 32.8 ± 0.33 | -1.35 ± 0.10 |
| [CO_2_] | **** | **** | ns |
| H_2_O | ns | ns | ns |
| T | ns | ns | ** |
| [CO_2_]*H_2_O | + | + | - |
| [CO_2_]*T | - | - | + |
| H_2_O*T | - | - | - |

The effects of Carbon Dioxide Concentration ([CO_2_]), water availability (H_2_O), temperature (T) and their peer interactions ([CO_2_]*H_2_O; [CO_2_]*T and H_2_O*T) have been performed by (one and two-way) Anova test using SPSS software. Significant effects are shown with asterisks (*, *P* ≤ 0.1; **, *P* ≤ 0.05; ***, *P* ≤ 0.01; ****, *P* ≤ 0.001; interaction between factors, +; no interaction between factors, -). Letters denote, ns, no significant differences (n=3).

**Table S2**. **Responsiveness of root C-N-related parameters from 60-days-old nodulated-alfalfa plants exposed to different climate change conditions.** Parameters: N content (%, m/m), C/N ratio and N natural isotopic signature (‰) of roots. Environmental conditions: CO_2_ concentration (400 or 700 µmol CO_2_ mol^-1^), temperature (ambient, Amb, or +4ºC) and water availability (well watered, WW, or water deficiency, WD). Data represent average values±SE (n = 3).

| Treatments | N Content | C/N | δ^15^N |
| --- | --- | --- | --- |
| (CO_2_-H_2_O-T) | (%) |  | (‰) |
| 400 - WW - Amb | 1.7 ± 0.06 | 21.0 ± 0.52 | -0.86 ± 0.08 |
| 400 – WD - Amb | 1.7 ± 0.07 | 17.0 ± 0.45 | -0.73 ± 0.04 |
| 400 - WW - +4ºC | 1.9 ± 0.11 | 18.7 ± 0.55 | -0.40 ± 0.04 |
| 400 - WD - +4ºC | 1.8 ± 0.14 | 19.1 ± 0.56 | -0.77 ± 0.02 |
| 700 - WW - Amb | 1.7 ± 0.06 | 23.7 ± 0.56 | -0.95 ± 0.06 |
| 700 – WD - Amb | 1.7 ± 0.05 | 20.0 ± 0.38 | -0.81 ± 0.05 |
| 700 - WW - +4ºC | 1.7 ± 0.10 | 16.1 ± 0.96 | -0.69 ± 0.06 |
| 700 - WD - +4ºC | 1.9 ± 0.10 | 19.4 ± 1.03 | -0.77 ± 0.02 |
| [CO_2_] | ns | ns | * |
| H_2_O | ns | ns | ns |
| T | ** | * | ** |
| [CO_2_]*H_2_O | - | - | - |
| [CO_2_]*T | - | + | - |
| H_2_O*T | - | + | + |

The effects of Carbon Dioxide Concentration ([CO_2_]), water availability (H_2_O), temperature (T) and their peer interactions ([CO_2_]*H_2_O; [CO_2_]*T and H_2_O*T) have been performed by (one and two-way) Anova test using SPSS software. Significant effects are shown with asterisks (*, *P* ≤ 0.1; **, *P* ≤ 0.05; ***, *P* ≤ 0.01; ****, *P* ≤ 0.001; interaction between factors, +; no interaction between factors, -). Letters denote, ns, no significant differences (n=3).

**Table S3.** **Responsiveness of nodule C-N-related parameters from 60-days-old nodulated-alfalfa plants exposed to different climate change conditions.** Parameters: N content (%, m/m), C/N ratio and N natural isotopic signature (‰) of nodules. Environmental conditions: CO_2_ concentration (400 or 700 µmol CO_2_ mol^-1^), temperature (ambient, Amb, or +4ºC) and water availability (well watered, WW, or water deficiency, WD). Data represent average values±SE (n = 3).

| Treatments | N Content | C/N | δ^15^N |
| --- | --- | --- | --- |
| (CO_2_-H_2_O-T) | (%) |  | (‰) |
| 400 - WW - Amb | 7.3 ± 0.22 | 5.7 ± 0.16 | -0.90 ± 0.03 |
| 400 – WD - Amb | 6.2 ± 0.57 | 5.7 ± 0.21 | -1.12 ± 0.06 |
| 400 - WW - +4ºC | 6.7 ± 0.09 | 5.7 ± 0.01 | -1.09 ± 0.00 |
| 400 - WD - +4ºC | 7.1 ± 0.32 | 5.5 ± 0.16 | -1.28 ± 0.01 |
| 700 - WW - Amb | 6.4 ± 0.40 | 5.9 ± 0.18 | -0.81 ± 0.01 |
| 700 – WD - Amb | 6.7 ± 0.09 | 6.1 ± 0.04 | -1.09 ± 0.05 |
| 700 - WW - +4ºC | 5.1 ± 0.21 | 6.2 ± 0.08 | -0.89 ± 0.03 |
| 700 - WD - +4ºC | 7.2 ± 0.57 | 5.9 ± 0.32 | -0.68 ± 0.14 |
| [CO_2_] | ns | *** | *** |
| H_2_O | ns | ns | ns |
| T | ns | ns | ns |
| [CO_2_]*H_2_O | - | - | - |
| [CO_2_]*T | - | - | + |
| H_2_O*T | - | - | - |

The effects of Carbon Dioxide Concentration ([CO_2_]), water availability (H_2_O), temperature (T) and their peer interactions ([CO_2_]*H_2_O; [CO_2_]*T and H_2_O*T) have been performed by (one and two-way) Anova test using SPSS software. Significant effects are shown with asterisks (*, *P* ≤ 0.1; **, *P* ≤ 0.05; ***, *P* ≤ 0.01; ****, *P* ≤ 0.001; interaction between factors, +; no interaction between factors, -). Letters denote, ns, no significant differences (n=3).

**Figure S1.** Scheme representing temperature gradient greenhouse (TGG) size, module distributions and gradient of temperature. Detail of heaters located in module 3 are shown in the picture.

**Figure S2.** Response of gas exchange parameters to differing conditions of CO_2_ concentration (400 µmol CO_2_ mol^-1^, left panels, or 700 µmol CO_2_ mol^-1^, right panels), temperature (ambient or +4ºC) and water availability (well watered, WW, or water deficient, WD) in 60-day-old nodulated alfalfa plants, measured at 400 (black plots) or 700 (white plots) µmol CO_2_ mol^-1^. Legend: (a and b) - leaf photosynthesis, *A*; (c and d) - transpiration, *Tr*; (e and f) - stomatal conductance, *g*; C_i_ - internal CO_2_ concentration. Data represent average values ± SE (n = 3-4).
